# MRSamePopTest: introducing a simple falsification test for the two-sample mendelian randomisation ‘same population’ assumption

**DOI:** 10.1186/s13104-024-06684-0

**Published:** 2024-01-17

**Authors:** Benjamin Woolf, Amy Mason, Loukas Zagkos, Hannah Sallis, Marcus R. Munafò, Dipender Gill

**Affiliations:** 1 School of Psychological Science, University of Bristol, Bristol, UK; 2 MRC Integrative Epidemiology Unit, University of Bristol, Bristol, UK; 3 MRC Biostatistics Unit, University of Cambridge, Cambridge, UK; 4 Victor Phillip Dahdaleh Heart and Lung Research Institute, University of Cambridge, Cambridge, UK; 5 British Heart Foundation Cardiovascular Epidemiology Unit, Department of Public Health and Primary Care, University of Cambridge, Cambridge, UK; 6 Department of Epidemiology and Biostatistics, School of Public Health, Imperial College London, London, UK; 7 Centre for Academic Mental Health, Population Health Sciences, Bristol Medical School, University of Bristol, Bristol, UK

**Keywords:** Sensitivity analysis, Two-sample Mendelian randomisation, Population homogeneity

## Abstract

Two-sample MR is an increasingly popular method for strengthening causal inference in epidemiological studies. For the effect estimates to be meaningful, variant-exposure and variant-outcome associations must come from comparable populations. A recent systematic review of two-sample MR studies found that, if assessed at all, MR studies evaluated this assumption by checking that the genetic association studies had similar demographics. However, it is unclear if this is sufficient because less easily accessible factors may also be important. Here we propose an easy-to-implement falsification test. Since recent theoretical developments in causal inference suggest that a causal effect estimate can generalise from one study to another if there is exchangeability of effect modifiers, we suggest testing the homogeneity of variant-phenotype associations for a phenotype which has been measured in both genetic association studies as a method of exploring the ‘same-population’ test. This test could be used to facilitate designing MR studies with diverse populations. We developed a simple R package to facilitate the implementation of our proposed test. We hope that this research note will result in increased attention to the same-population assumption, and the development of better sensitivity analyses.

**Supplementary Information:**

The online version contains supplementary material available at 10.1186/s13104-024-06684-0.

## Key message

• Two-sample Mendelian randomisation (2SMR) can be used to estimate the lifetime effect of a modifiable exposure on an outcome of interest.

• 2SMR point estimates are not interpretable if the exposure and outcome GWASs do not come from homogeneous populations, so called ‘same population’ assumption. However, this assumption is often not validated in applied studies.

• We propose and validate a novel sensitivity analysis for this assumption, which checks if SNP effects for the same trait are homogeneous across the two populations.

## Introduction

Mendelian randomisation (MR) is a natural experiment that leverages the independent and random inheritance of genetic variants to justify the assumptions of the instrumental variable (IV) framework [1–3]. Within this framework, genetic variants known to associate with an exposure of interest can be used to examine if an exposure of interest causes an outcome . Two-sample MR (2SMR) applies this approach using summary statistics from genome-wide association studies (GWASs). Advantages of 2SMR include greater statistical power, and the opportunity to apply estimators, like MR-Egger, that do not require all variants to be valid instruments [4]. However, 2SMR requires two additional assumptions: (1) that there is no sample overlap between the exposure and outcome GWAS, and (2) that the GWASs were sampled from the same population, or separate populations that are sufficiently similar that they can be treated as the same population.

The primary effect of the no-overlap assumption is to force weak instrument bias to attenuate results towards the null [4]. If the variants are strongly associated with the exposure (such as when the conventional *p* < 5 × 10^− 8^ threshold has been used to select instruments), the amount of weak instrument bias should be very small. Violations of this assumption are thus unlikely to be a serious threat to the internal validity of an MR study.

The same-population assumption has received less attention, but is still important. If the effect estimates are drawn from heterogeneous populations, then the interpretation of the MR estimate becomes unclear. When the GWASs do not have overlapping samples, the same-population assumption is generally addressed by exploring study demographics like age, sex, and ancestry [5]. However, this may not be sufficient because less easily accessible factors, such as the prevalence of smoking for a lung cancer MR study, may also be important. Other proposals, like comparing the GWASs’ allele frequencies as a test of homogeneous ancestry [6], also cannot detect if more subtle differences are important. Better ways to test the same-population assumption are therefore needed.

Methodological developments in the field of causal inference are being applied to investigate the generalisability of effect estimates. For example, Pearl developed the Data Fusion Framework as a “theoretical solution” to questions about the external validity of study estimates [7, 8]. Likewise, the Potential Outcomes framework can be modified to aid inference about generalisability and transportability [9–12]. These frameworks both postulate that we can generalise an estimate once there is an equivalence of factors, such as effect modifiers or selection effects, which would cause differences in the effect estimates between the study and target populations.

These frameworks could in theory be used to ensure that the estimates from one GWAS can generalise to another [13]. However, it is likely to be difficult (or impossible) to apply in genuine summary data settings where researchers do not have access to individual level data. For example, the Potential Outcomes framework requires knowing what all the relevant effect modifiers are, and the differences in the prevalence of these between the studies. However, Genome Wide Interaction Studies [14], and other GWAS-type studies which include interactions, are much rarer than GWASs, and are more likely to be underpowered. Researchers are therefore likely to struggle to ascertain all relevant effect modifiers. In addition, GWASs generally do not present sufficient demographic data to make this type of procedure possible for factors other than age, sex and ethnicity [15].

The randomised controlled trial (RCT) and meta-analysis literature have also introduced methods for combining estimates from different populations. Randomised controlled trials which have recurred people from different (sub-)populations, for example a multi-centre trial like the CRASH-II trial [16, 17], generally account for population differences by controlling for retirement centre in the analysis [18, 19]. The analogue for meta-analyses is a multi-level meta-analysis in which known population differences between trials are modelled by adding a random effect to the analysis model [20]. However, as with the previous frameworks, these methods are difficult to apply to 2SMR. For example, given that MR studies would typically be comparing effects from only two studies, they would lack the degrees of freedom to implement a multilevel meta-analysis. It therefore appears that existing methods for combining estimates from different populations would be difficult to apply in their current form to an MR setting.

The above methods all agree that two studies can be treated as coming from the same populations if their effect estimates are homogeneous. It follows that the same-population assumption can be tested by estimating the heterogeneity in the SNP effect estimates for a phenotype that has been measured in both samples. When the difference between two effect estimates on the same scale is zero, they are more likely homogeneous. Hence, we propose testing if the difference in the SNP-phenotype association(s) between the exposure and outcome sample is equal to zero as an easy-to-implement test of this assumption.

## Main text

Here we introduce a simple falsification test for the 2SMR ‘same population’ assumption. Our proposed test involves testing if the (average) SNP effect for a relevant phenotype is homogeneous between the two samples being used in the analysis. Although this could be implemented in multiple ways, a simple implementation is to test if the difference in the SNP effect estimates from the two samples is equal to zero for the SNP(s) used in the MR analysis. When multiple independent SNPs are used, the test can be implemented by meta-analysing the differences for each SNP (see the Supplement for more details). Where a difference is detected, that could be taken as evidence for a difference in the prevalence of effect modifiers (or another factor) between the two samples and hence, the effect estimates in one population will not generalise to another.

This test requires that at least one phenotype has been measured in both samples. We would suggest that when both samples have information on the exposure and outcome, the falsification test should be implemented on both phenotypes to provide reassurance that all potential effect modifiers are the same, and both average causal estimates (SNP-exposure and SNP-outcome) are homogeneous. If the datasets only have information on one of the phenotypes then the test should be performed using this phenotype. This assumes that the effect modifier(s) are the same for the unmeasured phenotype, which may not always be true. The test can also be performed when the samples have measured a common phenotype that is not the exposure or outcome. Applying this test to a non-exposure/outcome phenotype requires the assumption that this phenotype has the same effect modifiers as the exposure and/or outcome. This is a strong assumption, and careful thought is needed in choosing which phenotype(s) to use. The availability of data from broadly phenotyped cohort studies, like the UK Biobank, should enable the application of this method.

In the Supplement we present a theoretical intuition, as well as a simulation to test the validity of our method. The simulation finds that our falsification test generally correctly detected differences in the SNP effects unless the difference in the average treatment effect between the samples and the variance explained by the instrument was very small, difference ≤ 5% and variance ≤ 1% (Table 1). However, the false positive rate did increase as the variance explained by the instruments increased. Although this increase was small and does not happen when meta-analyzing multiple SNPs (Supplementary Table 1), it is thus perhaps due to chance given only 1000 iterations.

As an applied example, we compare the defences between GIANT and UK Biobank (UKB) weight GWASs. As a negative control, we did not expect to observe a difference between these two samples genetic associations for adult weight. When both were measured on the same scale (Kg) we did not observe a difference (Table 2), but we did when the UKB used a standard deviation scale instead. This shows the importance of ensuring that effect estimates are on the same scale. As a positive control, we compared the association between genetic associations for adult weight and birthweight, since variant-weight is known to vary with age, as a positive control [21]. We found that there were different effects between the genome-wide significant SNPs for adult weight and birthweight (Table 2).

## Limitations

A major limitation of all falsification tests is that, while they can provide evidence against an assumption, they cannot necessarily provide evidence to support it. However, the test can also produce misleading evidence of differences.

We showed in our supplementary simulation that different amounts of (residual) bias between GWASs, such as from population structure, can result in the detecting differences even when the GWASs use the same underlying population. This could theoretically create issues when using data from GWAS consortia which meta-analysed smaller studies. Since not all consortia force each study to perform identical GWASs, it could be difficult to compare the methodology to a single study GWAS. However, our applications of this method here and elsewhere to date imply that in practice consortia which use different methods to a single study GWAS, or which do not enforce homogenous methods, do not produce heterogeneous effects from single study GWASs drawn from a comparable population [22–24]. We would however suggest, when possible, triangulating our proposed sensitivity analysis with other approaches, such as a comparison of the measured demographic factors. Likewise, if two GWASs for the same phenotype have different covariates, then a difference in effect estimates could represent the effects of different amounts of collider bias (e.g. if only one GWAS has adjusted for a heritable phenotype such as BMI) or non-collapsibility issues in the case of odds ratios. Finally, differing levels of measurement error could also result in different effect estimates between even when the underlying populations are homogeneous.

If the same sample is used to choose genetic variants used in the test and estimate effects used for one of the populations, then this may create inflation (Winner’s curse) in this population but not in the other population. Hence the likelihood of a false positive (but not a false negative) might be higher in this setting. However, since we employed exactly this procedure in our applied examples, this bias may not be substantial in practice. This conclusion is supported by a recent simulation, which found that Winner’s curse introduced negligible amounts of bias for genome-wide significant SNPs in UK Biobank-sized GWASs [25, 26].

Three additional, but important, caveats need to be considered. Firstly power: because SNP effect estimates are often imprecise, this test may be underpowered. As with MR studies, power can sometimes be increased by including more SNPs that are less strongly associated with the exposure. However, including SNPs not used in the MR analysis will require assuming that these SNP’s effects are themselves homogeneous to those used in the MR analysis. In addition, if the SNP effect estimates are less precise, adding them could add noise and reduce power. Second, as illustrated in our applied example, our method requires that each GWAS measures effects with the same units. Finally, as with 2SMR, our proposed test requires that the SNP effect alleles between the GWASs have been harmonised.

Here we have focused on the use of MR for effect estimation. An alternative approach is to use MR to test the null hypothesis [27]. Testing for homogeneity is unnecessarily stringent when the MR study is only testing the null hypothesis. However, a monotonic version of the same population assumption is still needed. At an extreme, a study interested in the effects of alcohol consumption on cardiovascular disease which extracts variant-outcome associations from a GWAS in a population who do not drink will find a null MR association even if there is are strong variant-exposure associations in an exposure GWAS from a population who drink.

## Conclusions

Our proposed test allows researchers to assess the same-population assumption when the GWASs come from subtly different populations . For example, when using a multi-sex exposure GWAS, like smoking, with a sex-specific outcome, like complications during pregnancy. In addition, because our method does not require knowledge of specific effect modifiers, it is robust to issues relating to unmeasured covariate. Although the test cannot prove the assumption and will therefore often be sub-optimal, we hope that this research note will result in increased attention to the same-population assumption, and prompt the development of better sensitivity analysis.

**Table 1:** Accuracy of method for correctly testing for the presence of different levels of effect modification over 1,000 iterations. This simulation explored the use of the test to detect differences between a single sex GWAS and a mixed-sex population GWAS for a single instrument. The simulation therefore emulates settings where the outcome GWAS has been measured in a specific sex (e.g. male fertility) but where the explore need not be sex specific (e.g. genetically predicted PDE5 levels) [24]. Accuracy in the 0% change in effect setting represents the percentage of iterations in which the test fails to detect a difference. In all other settings it represents the percentage of iterations in which the test detects a difference. Similar results were found in a simulation with many SNPs (Supplementary Table 1)

Change in average effect between samples^1^	Expected variance explained by instrument^2^		
	10%	5%	1%
**50%**	100%	100%	100%
**37.5%**	100%	100%	100%
**25%**	100%	100%	100%
**12.5%**	100%	100%	100%
**5%**	100%	100%	57%
**2.5%**	100%	70%	19%
**0%**	93%	94%	95%

^1^ The mixed-sex GWAS had on average 50% of the sample from each sex. Thus a change in effect of 50% between the two samples means one sex has an effect that is a 100% larger the other (i.e. the rows are half the value of ‘EM’ in the supplement)

^2^ The expected variance explained by instrument is derived from the ε term in the simulation

**Table 2:** Results of the applied analysis comparing GIANT and UKB weight GWASs. GIANT = the 2013 Genetic Investigation of ANthropometric Traits consortia GWAS [28]. UKB = Ben Ellsworth UK Biobank GWASs [15]. GWS = genome wide significant (*p* < 5 × 10^− 8^)

GWAS 1	GWAS 2	p-value of meta-analysed difference between the 11 GWS GIANT SNPs		Interpretation
		Fixed effects	Fisher’s method	
GIANT weight (Kg)	UKB weight (Kg)	0.130	0.387	Since these are similar phenotypes in simpler populations, we should not, and do not, observe a difference in effect between the two GWASs
	UKB weight (SD)	< 0.001	< 0.001	When the GWASs are measured using different scales, we can misleadingly detect a difference between them
	UKB birthweight (Kg)	< 0.001	< 0.001	Variant-weight associations are known to change with age. As expected, we therefore observe a difference in associations between adult weight and birthweight

### Electronic supplementary material

Below is the link to the electronic supplementary material.

Supplementary Material 1

## Data Availability

We developed the MRSamePopTest R package (available from https://github.com/bar-woolf/MRSamePopTest/wiki) to facilitate the implementation of this falsification test. Please note that the current version assumes that variants are independent of each other. The code used in the applied example and simulation is available form 10.17605/OSF.IO/GYXTJ.
