# Supplementary material for: MRSamePopTest: introducing a simple falsification test for the two-sample mendelian randomisation ‘same population’ assumption
Source: BMC Res Notes. 2024 Jan 17;17:27. doi: 10.1186/s13104-024-06684-0 (PMC10795421; doi:10.1186/s13104-024-06684-0)
Supplement: Supplementary file 1 — Supplementary Material 1 [file 13104_2024_6684_MOESM1_ESM.docx]

**Supplement**

**Theoretical intuition**

Suppose we have an exposure or outcome, O, in population 1, which is caused by a binomially distributed genetic variant. The GWAS estimates the causal effect of a variant such that:

1. $\hat{\beta}_{1}= \beta_{1}+\varepsilon_{1}$

Where$\beta_{1}$is the true causal effect for the variant in population 1, $\hat{\beta}_{1}$is the effect estimate for the variant from a GWAS, $\varepsilon_{1}$is normally distributed error term with a mean of zero.

Likewise, let the effect estimate of the variant on O in the second population 2:

1. $\hat{\beta}_{2}= \beta_{2}+\varepsilon_{2}$

Where$\beta_{2}$ is the true causal effect for the variant in population 2, $\hat{\beta}_{2}$ is the effect estimate for the variant from a GWASs, $\varepsilon_{2}$ is normally distributed error term with a mean of zero.

If the causal effect of the genetic variant on O in population 1 generalises to population 2, then there is the same effect for the two variants (i.e. $\beta_{1}$= $\beta_{2}).$ All else being equal, it follows that if the two populations are comparable, then ${\hat{\beta}_{1}- \hat{\beta}}_{2}=0$. Hence, if a test of the difference between $\hat{\beta}_{1}$ and $\hat{\beta}_{2}$ finds a difference between the two, then this implies that the estimates are not comparable.

If there is a difference in the prevalence of an effect modifier, then the strata specific of $\beta_{1}$ and $\beta_{2}$ can be identical, but the average causal effect estimated by the GWASs for the two populations will differ. Advocates of the Potential Outcomes framework have suggested this as a requirement for effect estimates to generalise (i.e. $\hat{\beta}_{1}$ will generalise to $\hat{\beta}_{2}$ when there is exchangeability of effect modifiers). For simplicity, imagine a binary effect modifier, like sex. The unstratified effect estimates will now be:

1. $\hat{\beta}_{1}=w_{F}*\beta_{1}*{EM}_{F}+w_{M}*\beta_{1}*{EM}_{M} +\varepsilon_{1}$
2. $\hat{\beta}_{2}=w_{F}*\beta_{2}*{EM}_{F}+w_{M}*\beta_{2}*{EM}_{M} +\varepsilon_{2}$

Where $w_{F}$ is the prevalence of females in the population, $w_{M}$ is the prevalence of males in the population, and ${EM}_{M}$ and ${EM}_{F}$ are the effect modifications for being male or female, respectively. Therefore, when $w_{F}$ $\neq$ $w_{M}$, then ${\hat{\beta}_{1}- \hat{\beta}}_{2}\neq0$, so a test for the difference between $\beta_{1}$ and $\beta_{2}$ should detect when the Potential Outcomes framework suggests the effects are not comparable.

From these two examples, it would seem that when there is a difference between $\hat{\beta}_{1}$ and $\hat{\beta}_{2}$ (i.e. ${\hat{\beta}_{1}- \hat{\beta}}_{2}\neq0$), then the two GWASs do not come from comparable populations. However, suppose there is some bias, $\alpha$, in the estimate of O such that:

1. $\hat{\beta}_{1}= \beta_{1}+ \alpha_{1}+ \varepsilon_{1}$
2. $\hat{\beta}_{2}= \beta_{2}+ \alpha_{2}+ \varepsilon_{2}$

It follows that when $\alpha_{1} \neq\alpha_{2}$, ${\hat{\beta}_{1}- \hat{\beta}}_{2}\neq0$ even if $\beta_{1}$= $\beta_{2}$. So, if the GWASs suffer from different amounts of bias, a test of the difference in effect estimates may reject the null hypotheses despite the populations being homogeneous. Because Mendel’s laws of inherence provide some robustness against most epidemiological biases, and since most GWASs use similar methods to control for bias, we would expect that in practice $\alpha_{1}\approx\alpha_{2}$.

Thus far we have only considered settings with a single SNP. However, most MR analyses use many SNPs. If the IV assumptions are valid, then we should expect all the SNP-specific Wald ratios to be estimating the same quantity. This intuition is widely used to motivate ‘robust’ estimators (1). Under a similar logic, the comparative effect sizes in two GWAS should be the same for each SNP. An intuitively sensible implementation is to meta-analyses the SNP-specific differences.

One way of doing this would be to perform an inverse variance weighted (IVW) meta-analysis of the differences. By doing so, this approach account for differences in precision of the differences in different SNPs effects. However, IVW is a signed meta-analysis. We suggest ensuring that all the effects of the first GWAS are positive so that negative differences all represent the second GWAS having smaller or discordant effects than the first and positive differences represent a stronger effect in the same direction in the second GWAS. To illustrate the issue, imagine two SNPs in the first GWAS with similar size effects but one is positive and the other negative, and for simplicity assume the effects size is zero in the second GWAS. The differences would in this instance sum to zero despite there being meaningful differences in effect sizes between the two GWASs. However if the SNP effects were recoded so that all of the effects for the first GWAS were all positive, the combined sum would show a large difference. Unlike looking at the absolute defences, orientating all exposure associations to be positive should not result in inflated type-1 errors.

An alternative approach would be to do the meta-analyses using Fisher’s method (2). This approach simply combines the p-values. It is therefore robust to directionality of the differences in SNP effects, since p-values are not signed.

This method assumes that SNPs are independent and are therefore no in linkage disequilibrium (LD) with each other. The inverse variance weighted meta-analysis of the differences could be extended to account for LD along the lines to the two-sample MR IVW estimator (3). An alternative paradigm which we do not explore further is to test if the ratio of the SNP effects is different from 1, rather than if the absolute difference is zero. The SNP effects could then be combined using an approach similar to the MR-IVW estimator, although it would be inappropriate to use the first order Delta method approximation of the standard error of the ratio.

Finally, we could in theory use reasoning like that above to develop a method to correct the estimates for the violations of the same population assumption. Such a method might be analogous to those already used to correct MR estimates for collider bias (4–6).

**Simulation**

*Aim*s: We ran a simulation to validate the proposed test as a method for falsifying the same population assumption. In this setting, there is a single sample in which the outcome has been measured, but the exposure is measured both in this sample (the outcome sample) and an independent sample (the exposure sample). The motivation in this simulation is a setting like our study proxying the effect of Viagra on male fertility where we want to maximise power in the exposure GWAS by using a whole population GWAS (of blood pressure) instead of sex-specific (blood pressure) GWAS (7). Doing so requires that the variant-exposure associations are similar in the male only GWAS to the combined sex GWAS. We report our simulations using the ADEMP (aims, data-generating mechanisms, estimands, methods, and performance measures) approach (8):

*Data-generating mechanisms*: We simulated a single nucleotide polymorphism (SNP) as two identically distributed one-level binomial distributions:

$SNP=B\left( 0.5, 1 \right)+B(0.5, 1)$

We then simulated a phenotype, P, in a male only GWAS with 250,000 participants as:

$P_{m1}=0.5*SNP+ \varepsilon_{1}$

$P_{m2}=0.5*SNP+ \varepsilon_{2}$

$P_{m3}=0.5*SNP+ \varepsilon_{3}$

Where ε_1_, ε_2_ and ε_3_ are a normally distributed error term such that ε_1_ ~ N(0, 1.05), ε_2_ ~ N(0, 1.53) and ε_3_ ~ N(0, 3.5). The variances were selected so that SNP explains around 0.1, 0.05, and 0.01% of the variance in the phenotype

We then simulated an independent mixed sex population GWAS with 700,000 participants where the phenotype was defined as:

$P_{p1}=0.5*SNP+0.5*EM*SNP*FEM+ \varepsilon_{1}$

$P_{p2}=0.5*SNP+0.5*EM*SNP*FEM+ \varepsilon_{2}$

$P_{p3}=0.5*SNP+0.5*EM*SNP*FEM+ \varepsilon_{3}$

Where FEM is a binomially distributed effect modifier in the outcome sample to represent participants being female, such that FEM ~ B(0.5, 1). EM is the relative change in effect between males and females due to sex based effect modification, and could take the values of 2, 1.75, 1.5, 1.25, 1.1, and 1.05.

*Estimand:* The p-value for the difference in the effect estimates for the exposure between the exposure and outcome samples.

*Methods*: In an analogy to a Genome-Wide Association study, we estimated the main effect of a linear regression of each exposure (separately) on the SNP. The difference in the effects between the GWASs was then tested as by subtracting one effect estimate from the other, and the standard error was estimated using the propagation of error method.

*Performance measure:* The accuracy of the test, defined as the proportion of iterations in which the test correctly identifies a difference in the SNP effects. Correct identification is operationalised as a p-value < 0.05 when there is a true difference in SNP effects, and p-value > 0.05 when there is not. This was estimated over 1,000 iterations. For the primary simulation, we also look at the mean estimated difference between the two samples across the iterations.

*Additional simulations*

Firstly, we explored the robustness of the test to different amounts of bias in the GWASs, even when there is no difference in effect modifiers. Specifically we explore the effect of residual/uncontrolled potential population structure in a GWAS in the second sample.

To do this, we simulated a second confounder U such that, it is a truncated normal distribution with a mean of 0.5, a standard deviation of 0.15, a maximum of 1 and a minimum of 0. We then let U determine both the allele frequency of the SNP and influence the distribution of the phenotype:

$${SNP}_{U}=B\left( U, 1 \right)+B(U, 1)$$

$$P_{U}=0.5*{SNP}_{U}+U+ \varepsilon_{1}$$

For simplicity, we assumed that there was no sex-based effect modification in this setting. We depict this setting visually using a Directed Asycicl Graph in Supplementary Figure S1.

Secondly, to explore the performance of the meta-analysis we replicated the simulation without residual confounding, but replaced SNP with an array of 15 independently and identically distributed SNPs each with the same definition as “SNP” in the original simulation, and the same effect on the phenotype. We then meta-analysed the SNP specific effects using both Fisher’s method and an inverse variance weighted meta-analysis of the SNP-specific differences, and explored the accuracy of the two methods. In addition, $\varepsilon_{1}$, $\varepsilon_{2}$, and $\varepsilon_{3}$were updated to be N(0, 4.1), N(0, 6), and N(0, 13.5) respectively in order to maintain the desired r^2^ while accounting for the additional variation in the phenotype.

*Additional Simulation results*

In the setting in which the second GWAS suffers from residual population structure (and therefore has inflated estimates), but there is no difference in the true effects between the samples, our test incorrectly rejected the null hypothesis in 100% of iterations.

The results of the simulation using many SNPs can be found in Supplementary Table S1. These imply that both meta-analysis methods are correctly deciding that there is a difference more than 90% of the time when there is only a 12.5% difference in the estimates between samples and the instruments together only explain 1% of the overall phenotypic variation. In the settings with no difference, the tests seemed to be rejecting the null hypothesis around the expected 5% rate.

Supplementary Table S2 provides the mean absolute difference, and standard deviation, between the two samples in the primary sample. These imply that the method is on average correctly estimating the differences between the two samples.

| **Relative change in average effect between samples^1^** | **Expected variance explained (r^2^) by instrument^2^** | | | | | | | | |
| --- | --- | --- | --- | --- | --- | --- | --- | --- | --- |
|  | **10%** | | | **5%** | | | **1%** | | |
|  | **Fisher** | **IVW** | **Fisher** | | **IVW** | **Fisher** | | **IVW** |  |
| **50%** | 100% | 100% | 100% | | 100% | 100% | | 100% |  |
| **37.5%** | 100% | 100% | 100% | | 100% | 100% | | 100% |  |
| **25%** | 100% | 100% | 100% | | 100% | 100% | | 100% |  |
| **12.5%** | 100% | 100% | 100% | | 100% | 98% | | 100% |  |
| **5%** | 100% | 100% | 89% | | 100% | 20% | | 57% |  |
| **2.5%** | 54% | 92% | 25% | | 66% | 8% | | 20% |  |
| **0%** | 96% | 94% | 93% | | 94% | 95% | | 94% |  |

Supplementary Table S1: Accuracy in detecting differences between samples in simulation with many (n = 15) SNPs. Accuracy in the 0% change in effect setting represents the percentage of iterations in which the test fails to detect a difference. In all other settings it represents the percentage of iterations in which the test detects a difference.

^1^ The mixed-sex GWAS had on average 50% of the sample from each sex. Thus a change in effect of 50% between the two samples means one sex has an effect that is a 100% larger the other (i.e. the rows are half the value of ‘EM’ in the supplement).

^2^ The expected variance explained by instrument is derived from the ε term in the simulation.

| **Relative change in average effect between samples^1^** | **Expected variance explained (r^2^) by instrument^2^** | | |
| --- | --- | --- | --- |
|  | **10%** | **5%** | **1%** |
| **50%** | -0.250 (0.004) | -0.250 (0.005) | -0.250 (0.012) |
| **37.5%** | -0.188 (0.004) | -0.187 (0.005) | -0.187 (0.012) |
| **25%** | -0.0125 (0.004) | -0.125 (0.005) | -0.125 (0.011) |
| **12.5%** | 0.063 (0.004) | -0.062 (0.005) | -0.062 (0.012) |
| **5%** | -0.025 (0.004) | -0.024 (0.005) | -0.025 (0.011) |
| **2.5%** | -0.013 (0.004) | -0.012 (0.005) | -0.012 (0.012) |
| **0%** | 0.000 (0.004) | 0.000 (0.005) | 0.000 (0.011) |

Supplementary Table S2: Mean absolute difference (standard deviation) between the male only sample (effect = 0.5) and mixed sex sample estimated by the method over 1,000 iterations. Negative differences imply that the mixed sex effects are larger than the mixed-sex sample.

^1^ The mixed-sex GWAS had on average 50% of the sample from each sex. Thus a change in effect of 50% between the two samples means one sex has an effect that is a 100% larger the other (i.e. the rows are half the value of ‘EM’ in the supplement).

^2^ The expected variance explained by instrument is derived from the ε term in the simulation.


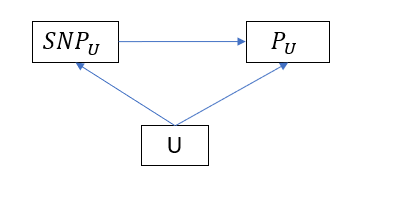


Supplementary Figure S1: Directed Acyclic Graph of simulation setup with population structure.

**Applied example**

As an applied example, we looked at the association between genetic variants and weight.

We extracted independent (clumping r^2^ = 0.001, clumping kb = 10,000) genome wide significant SNPs from the GIANT 2013 GWAS of weight (133,723 male and female adults; OpenGWAS IDs: ieu-a-106 (males), ieu-a-107 (females)). GIANT is a GWAS consortia which meta-analysed cohorts with European ancestry individuals (9). This produced a set of 11 SNPs. As a negative control, we compared the effects between the GIANT GWAS to a UK Biobank GWAS of adult weight (461,632 male and female European ancestry adults; OpenGWAS ID: ukb-b-11842). Since both GIANT and the UKB have measured adult weight in adults and have used similar European ancestry GWASs we would not expect to find large differences in effects between these two samples. We found proxies using TwoSampleMR when a SNP was missing in the second GWAS (r^2^ = 0.8)

We then explore the importance of using consistent units between the GWASs by reimplementing this analysis after rescaling the effects for weight in the UK Biobank to be standard deviation scale (whereas the previous analyses used unstandardized effects with units of Kg for both GWASs).

As a positive control, we therefore compared the difference in effect estimates between genome-wide significant SNPs from the GIANT 2013 GWAS and the MRC-IEU’s UK Biobank GWAS of birthweight (261,932 male and female adults self-reporting their own birthweight; OpenGWAS ID: ukb-b-1337) (9). Variant-weight association are known to vary with age (10). Unlike the first analysis, we expected to detect differences for this phenotype.

**References**

1. Slob EAW, Burgess S. A comparison of robust Mendelian randomization methods using summary data. Genetic Epidemiology. 2020;44(4):313–29.

2. Mosteller F, Fisher RA. Questions and Answers. The American Statistician. 1948;2(5):30–1.

3. Burgess S, Zuber V, Valdes-Marquez E, Sun BB, Hopewell JC. Mendelian randomization with fine-mapped genetic data: Choosing from large numbers of correlated instrumental variables. Genetic Epidemiology. 2017;41(8):714–25.

4. Dudbridge F, Allen RJ, Sheehan NA, Schmidt AF, Lee JC, Jenkins RG, et al. Adjustment for index event bias in genome-wide association studies of subsequent events. Nat Commun. 2019 Apr 5;10(1):1561.

5. Brown BC, Ye CJ, Price AL, Zaitlen N. Transethnic Genetic-Correlation Estimates from Summary Statistics. Am J Hum Genet. 2016 Jul 7;99(1):76–88.

6. Mitchell RE, Hartley AE, Walker VM, Gkatzionis A, Yarmolinsky J, Bell JA, et al. Strategies to investigate and mitigate collider bias in genetic and Mendelian randomisation studies of disease progression. PLOS Genetics. 2023 Feb 23;19(2):e1010596.

7. Woolf B, Rajasundaram S, Cronjé HT, Yarmolinsky J, Burgess S, Gill D. The association of genetically proxied sildenafil with fertility, sexual activity, and wellbeing: a Mendelian randomisation study [Internet]. medRxiv; 2023 [cited 2023 Oct 30]. p. 2023.03.27.23287822. Available from: https://www.medrxiv.org/content/10.1101/2023.03.27.23287822v1

8. Morris TP, White IR, Crowther MJ. Using simulation studies to evaluate statistical methods. Statistics in Medicine. 2019;38(11):2074–102.

9. Randall JC, Winkler TW, Kutalik Z, Berndt SI, Jackson AU, Monda KL, et al. Sex-stratified Genome-wide Association Studies Including 270,000 Individuals Show Sexual Dimorphism in Genetic Loci for Anthropometric Traits. PLOS Genetics. 2013 Jun 6;9(6):e1003500.

10. Sanderson E, Richardson TG, Morris TT, Tilling K, Smith GD. Estimation of causal effects of a time-varying exposure at multiple time points through multivariable mendelian randomization. PLoS Genet. 2022 Jul 18;18(7):e1010290.
